# Glycan Binding Profiling of Jacalin-Related Lectins from the *Pteria Penguin* Pearl Shell

**DOI:** 10.3390/ijms20184629

**Published:** 2019-09-18

**Authors:** Tomohisa Ogawa, Rie Sato, Takako Naganuma, Kayeu Liu, Agness Ethel Lakudzala, Koji Muramoto, Makoto Osada, Kyosuke Yoshimi, Keiko Hiemori, Jun Hirabayashi, Hiroaki Tateno

**Affiliations:** 1 Graduate School of Life Sciences, Tohoku University, Sendai 980-8577, Japan; riesato310@gmail.com (R.S.); naga@biochem.tohoku.ac.jp (T.N.); knight.lky@gmail.com (K.L.); lakudzala.agness.ethel.r3@dc.tohoku.ac.jp (A.E.L.); muramoto@biochem.tohoku.ac.jp (K.M.); 2 Center for Interdisciplinary Research, Tohoku University, Sendai 980-8578, Japan; makoto.osada.a8@tohoku.ac.jp (M.O.); yoshimik@fris.tohoku.ac.jp (K.Y.); 3 Graduate School of Agricultural Science, Tohoku University, Sendai 980-8572, Japan; 4 Department of Materials Science, Graduate School of Engineering, Tohoku University, Sendai 980-8579, Japan; 5 Biotechnology Research Institute for Drug Discovery, National Institute of Advanced Industrial Science and Technology, Tsukuba 305-8568, Japan; Keiko-hiemori@aist.go.jp (K.H.); jun-hirabayashi@aist.go.jp (J.H.); h-tateno@aist.go.jp (H.T.)

**Keywords:** biomineralization, chitin, glycan binding profiling, lectin, pearl shell

## Abstract

We determined the primary structures of jacalin-related lectins termed PPL3s (PPL3A, 3B, and 3C, which are dimers consisting of sequence variants α + α, α + β, β + β, respectively) and PPL4, which is heterodimer consisting of α + β subunits, isolated from mantle secretory fluid of *Pteria penguin* (Mabe) pearl shell. Their carbohydrate-binding properties were analyzed, in addition to that of PPL2A, which was previously reported as a matrix protein. PPL3s and PPL4 shared only 35–50% homology to PPL2A, respectively; they exhibited significantly different carbohydrate-binding specificities based on the multiple glycan binding profiling data sets from frontal affinity chromatography analysis. The carbohydrate-binding specificity of PPL3s was similar to that of PPL2A, except only for Man_3_Fuc_1_Xyl_1_GlcNAc_2_ oligosaccharide, while PPL4 showed different carbohydrate-binding specificity compared with PPL2A and PPL3s. PPL2A and PPL3s mainly recognize agalactosylated- and galactosylated-type glycans. On the other hand, PPL4 binds to high-mannose-and hybrid-type N-linked glycans but not agalactosylated- and galactosylated-type glycans.

## 1. Introduction

Biomineralization is the crystallization process of inorganic materials under strict biological control in nature. This process generates biominerals such as bones, teeth, eggshells, and shells. Such biominerals are organic–inorganic hybrid nano-composites composed of organic matrices, such as proteins, lipids, and polysaccharides, that function as templates or nucleation centers. Mother of pearl, also known as nacre, is one of the sophisticated organic–inorganic hybrid materials composed of aragonite tablets arranged in consecutive mineral lamellae, like a photonic crystal. Furthermore, pearl shell oysters can concurrently produce two distinguished types of calcium carbonate crystals: Calcite on the prismatic layer and aragonite on the nacreous layer. To date, several matrix proteins and biomineralization-related genes have been identified via transcriptome and proteome analyses [1,2,3,4,5,6,7,8]. Interestingly, matrix proteins include lectin and lectin-domain proteins such as C-type lectins, galectins [9], and F-type lectins [10]. In particular, C-type lectins have been widely identified in biominerals, such as pancreatic stone protein (lithostathine) [11], *Megabalanus rosa* (acorn barnacles) lectins (BRAs) [12], eggshell ovocleidin 17 [13], perlucin from *Halotis laevigata* (abalone) [8,14], and brittle stars [15,16]. Perlucin has been reported to enhance the precipitation of calcium carbonate in solution [17,18]. Recently, splice variants of perlucin, of which C-terminal region include repeat sequence, has been identified [19]. These diversifications of C-type lectins were also observed in the spicule matrix proteins of sea urchin, which include Pro-rich repeat domain and/or acidic repeat domain [20]. However, the relevance of these diverse lectins to biomineralization, especially their carbohydrate-binding abilities and biomineralization activities, are still not clear.

Lectins are proteins or glycoproteins of non-immune origin, and are non-enzymes that bind specifically and reversibly to carbohydrates, resulting in cell agglutination or precipitation via recognition of polysaccharides or glycoconjugates [21]. Lectins are ubiquitous in biosphere and have been found in viruses, bacteria, fungi, plants, and animals. One of the most probable roles of marine invertebrate lectins is considered to be humoral factors in defense system. Previously, we isolated an 18-kDa lectin, termed PPL1, as a biodefense molecule from the mantle of *Pteria penguin* large-winged pearl shells. PPL1 showed sequence homology with rhamnose-binding lectins (RBLs) from various fish eggs and a galactose-binding lectin (SUEL) from sea urchin eggs [22]. Furthermore, we also identified and characterized novel matrix proteins, PPL2A and PPL2B, which are dimers composed of α + γ and β + β subunits, respectively, and are homologous to jacalin-related-prism fold lectins, from *P. penguin* mantle and nacre [23]. PPL2A and PPL2B regulated the morphological populations of calcite crystals in vitro, suggesting that lectins from the secreted fluid of *P. penguin* are involved not only in biodefense, but also in shell formation [23]. In addition to PPL1 and PPL2s, unadsorbed fractions of mucin and trehalose-liganded affinity chromatographies retained hemagglutination activity, indicating that other novel lectin(s) exists in mantle fluid. Thus, the diversification of lectins in *P. penguin* mantle may be closely related to biomineralization, as well as C-type lectins such as parlucin and spicule matrix proteins. Further, we determined the three-dimensional (3D) structures of PPL3s by X-ray crystallography, including the free lectins (5YRE, 5YRH, 5YRK) and lectins in complex with trehalose (5YRF, 5YRI, 5YRL) and isomaltose (5YRG, 5YRJ, 5YRM) [24]. It is important to elucidate the carbohydrate-binding specificities of these lectins because recognition of carbohydrates is a key element of many processes in biological function, including biomineralization.

In this study, we determined the primary structures of two jacalin-related lectins, termed PPL3s and PPL4, which were isolated from the mantle-secreted fluid of *P. penguin*, and analyzed their carbohydrate-binding properties, in addition to that of PPL2A, in detail.

## 2. Results

### 2.1. Purification and Characterization of Novel Lectins from the Secretory Fluid of Pteria Penguin Mantle

**Figure 1:**
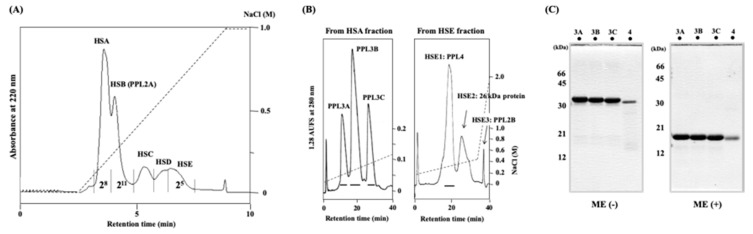
Purification of PPL3s and PPL4. Cation-exchange chromatography of the secreted fluid of *Pteria penguin* mantle on POROS HS column (**A**) and on Resource S column (ø 6.4 × 30 mm) (**B**). Flow rate was 1 mL/min. Lectin activity of each peak fraction in (**A**) was indicated by hemagglutination activity that defined as a titer value of maximum dilution with positive agglutination of 2% rabbit erythrocytes. SDS-PAGE (15%) profiles of PPL3s (3A, 3B, 3C) and PPL4 under non-reducing (ME(–)) and reducing (ME(+)) conditions after purification (**C**).

Secretory fluid of *P. penguin* mantle that contains lectin hemagglutinating activities was separated by two-dimensional ion-exchange chromatographies using combined POROS HS and Resource S columns. Figure 1A shows the typical chromatogram of *P. penguin* mantle secreted fluid proteins separated by cation-exchange chromatography on a POROS HS column, yielding five peaks: HSA, HSB, HSC, HSD, and HSE. Hemagglutinating (lectin) activities were detected in HSA, HSB, and HSE fractions, respectively. Western blot analysis using anti-PPL2A antibody and N-terminal amino acid sequence of purified proteins revealed that HSB fraction contained PPL2A previously identified [23]. Thus, HSA and HSE fractions that contained novel lectins were further purified by cation-exchange chromatography on a Resource S column. HSA contained three peaks, corresponding to PPL3A, 3B, and 3C. HSE fraction also yielded three peaks (HSE1–3). From SDS-PAGE and N-terminal sequence analyses, it was confirmed that peak fraction HSE1 contained novel lectin protein termed PPL4, HSE2 contained 26 kDa protein without lectin activity, and HSE3 contained PPL2B, which was previously reported [23] (Figure 1B). Figure 1C shows SDS-PAGE profiles of purified novel lectins PPL3A, 3B, 3C, and PPL4 that gave single bands under non-reducing and reducing conditions, corresponding to 32 kDa and 16 kDa, respectively. These results indicate that PPL3s and PPL4 exist as a dimer cross-linked by intermolecular disulfide bonds, respectively.

### 2.2. Amino Acid Sequences of PPL3 and PPL4 Subunits

Determination of the amino acid sequences of PPL3A, 3B, and 3C subunits was conducted by combining Edman sequencing using peptide mapping and cDNA sequencing. The peptide maps of PPL3A, 3B, and 3C obtained by digestion with *Achromobacter* protease I and *Staphylococcus aureus* V8 protease showed very similar profiles (Supplementary Materials Figure S1), suggesting that PPL3A, 3B, and 3C are iso-lectins with similar amino acid sequences. In fact, PPL3A contained V7, V8, and V14 fragments, which corresponded to the peptides NCIQWSKKG**E**, NCIQWSKKG**E**KVVHE, and YLGGPGGDAFDDKA**V**AQNGDITRIE, respectively, and PPL3C contained V10 and V16 peptide fragments corresponding to NCIQWSKKG**V**KVVHE and YLGGPGGDAFDDKA**L**AQNGDITRIE, respectively (Supplementary Materials Figure S1 and Table S1). On the other hand, PPL3B contained all of these fragments (V7, V8, V10, V14, and V16). These results suggest that PPL3A, 3B, and 3C are iso-lectins composed of two kinds of subunits (α and β); that is, α + α, α + β, and β + β, respectively. cDNAs encoding PPL3α and β subunits were composed of 483 bp with an open reading frame of 426 nucleotides, encoding PPL3 subunits consisting of 142 amino acid residues (Supplementary Materials Figure S2). The amino acid sequences of PPL3α and PPL3β deduced from cDNA sequences corresponded with those from peptide sequencing, except for N-terminal two amino acids, Gln-Val or Glu-Ile.

On the other hand, two subunits (α and β) of PPL4 were separated by rpHPLC after reduction and carboxamidomethylation of PPL4 (Supplementary Materials Figure S3), and it was determined their N-terminal amino acid sequences were SCGALSESYGGPGGLNRFDE… and VCTALSESYGGPGGLNRFDE…, respectively. Furthermore, the full-length amino acid sequences of PPL4α and β subunits were determined by Edman sequencing using peptide maps with *Achromobactor* protease I (Supplementary Materials Figure S4 and Table S2), as well as cDNA sequencing using the RACE method. Thus, cDNAs encoding α and β subunits of PPL4 included 510 bp with open reading frames of 444 nucleotides for a mature protein of 148 amino acid residues, respectively, and showed approximately 92% sequence identity with each other (Supplementary Materials Figure S5). In the previous study, we determined the disulfide bond patterns of PPL2A and PPL2B by MALDI Tof MS/MS analysis combined with peptide mapping, resulting two intrachain disulfide bonds, Cys37–Cys109 and Cys63–Cys134, and one interchain disulfide bond at N-terminal region [23].

**Figure 2:**
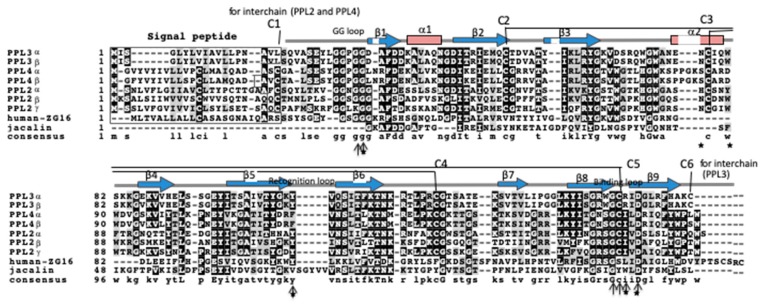
Aligned amino acid sequences of PPL3 and PPL4 subunits with jacalin-related lectins. The sequences were aligned using the Clustal W program [25] and represented by using BOXSHADE 3.21. The residues identical to the column-consensus are presented by inverse character (black background), while the residues which are not identical, but at least similar to the column-consensus, are presented by gray background. C1 to C6 indicates the half-cysteine residues with inter- and intra-disulfide bonds. Secondary structural elements, β strands (β1–β9) and α helixes (α1 and α2), identified in the 3D structure of PPL3s [24] are shown as blue arrows and red boxes, respectively. The GG loop, the recognition loop, and the binding loop, indicated by arrows, are parts of the carbohydrate recognition domain. Asterisk (*) indicates the amino acid residues taking part in the interaction with carbohydrates found in the three-dimensional (3D) structure of PPL3s.

**Figure 3:**
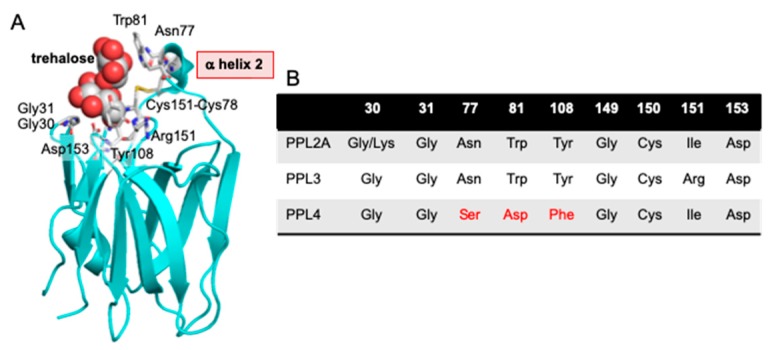
Schematic structure of PPL3 subunit conjugated with trehalose (**A**) and a comparison of the amino acid residues of carbohydrate recognition domains (CRDs) among PPL2A, PPL3s, and PPL4 (**B**). The 3D structure of PPL3s with trehalose (PDB ID 5yrf) was visualized by using Pymol 2.3 [26]. Trehalose ligands and amino acid residues involved in carbohydrate binding are indicated by spheres and sticks model, respectively.

Figure 2 shows the aligned amino acid sequences of PPL3 and PPL4 subunits, in addition to PPL2A and 2B subunits, jacalin and human ZG16 protein. From the sequence alignment for PPL3 and PPL4 subunits with PPL2s, the disulfide bond profiles of PPL3s and PPL4 were estimated to be two intrachain disulfide bonds (C2–C5 and C3–C4) and one interchain disulfide bond (C6–C6 for PPL3s or C1–C1 for PPL4), as shown in Figure 2. Recently, we also confirmed the disulfide bond patterns of PPL3s by their 3D structures determined by X-ray crystallography [24]. PPL2A, 2B, and PPL4 contain two intramolecular disulfide bonds and one interchain disulfide bond at N-terminus, while only PPL3s contain half-cystine for interchain disulfide bond at C-terminus, in addition to two intramolecular disulfide bonds (Figure 2). These results indicate PPL3s have different dimeric orientation (tail-to-tail) from other *Pteria penguin* lectins (PPLs) (head-to-head). The 3D structure of PPL3s revealed that the residues Gly31, Asn77, Trp81, Tyr108, Arg151, and Asp153 are in contact with carbohydrates (Figure 3). Among them, Gly31 at the GG loop and Asp153 at the carbohydrate-binding domain were conserved in all PPLs—PPL2A, PPL2B, PPL3s, and PPL4. On the other hand, the amino acid residues Asn77 and Trp81 on helix 2 and Tyr108 at the recognition loop were conserved between PPL2 and PPL3s, but were replaced by Ser77, Asp81, and Phe108 in PPL4, respectively (Figure 2 and Figure 3).

### 2.3. Carbohydrate-Binding Profilings of PPL2A, PPL3s, and PPL4

To investigate the sugar-binding specificities of PPL3s and PPL4, a hemagglutination inhibition assay using rabbit erythrocytes and several carbohydrates was conducted. The sugar-binding specificities of PPL3A, PPL3B, and PPL3C were identical (data not shown), and D-fructose, trehalose, and isomaltose were the most potent saccharide inhibitors for PPL3s—the same as PPL2A (Table 1). Conversely, PPL4 showed a broad specificity for saccharides, including to D-glucose, D-fructose D-mannose, D-glucosamine, trehalose, kojibiose, maltose, and *N*-acetyl-D-glucosamine (Table 1). In particular, PPL4 had high affinity for *N*-acetyl-D-glucosamine and its oligomer, chitin oligomers (*n* = 2–7). Asialofetuin showed strong inhibitory activities against all PPLs, while asialomucin (types I and II) and heparin showed no inhibitory activity.

In order to profile the carbohydrate-binding specificities of PPL3s and PPL4, in addition to PPL2A, more rigorously from a comprehensive viewpoint, we analyzed their sugar-binding properties against 130 pyridylaminated (PA) glycans, including 61 N-liked glycans and 39 glycolipid-type glycans (Supplementary Materials Figure S6) by the automated frontal affinity chromatography (FAC) system [27,28]. For evaluation of lectin-conjugated columns for FAC analysis, it was necessary to determine the effective ligand content (B_t_) based on the concentration-dependence analysis using Man3GlcNAc2 glycan-conjugated 9-fluorenyl methoxycarbonyl asparagine (1M2M-5NC-Asn (Fmoc)) for PPL2A and PPL3s, and 4-nitrophenyl-α-D-mannnopyranoside (Manα1-pNP) for PPL4, respectively. As a result, B_t_ and the equilibrium dissociation constant *(K*_d_) values were determined to be 0.02 nmol and 2.0 × 10^−7^ M, respectively, for PPL2A column, 0.63 nmol and 3.01 × 10^−5^ M for PPL3s column, and 0.98 nmol and 2.0 × 10^−5^ M for PPL4 column (Supplementary Materials Table S3). These results indicate that PPL2A showed stronger binding affinity (~100 times) to 1M2M-5NC-Asn than PPL3s. This strong binding ability of PPL2A compared with PPL3s and PPL4 was also detected for other glycans, as described later.

**Figure 4:**
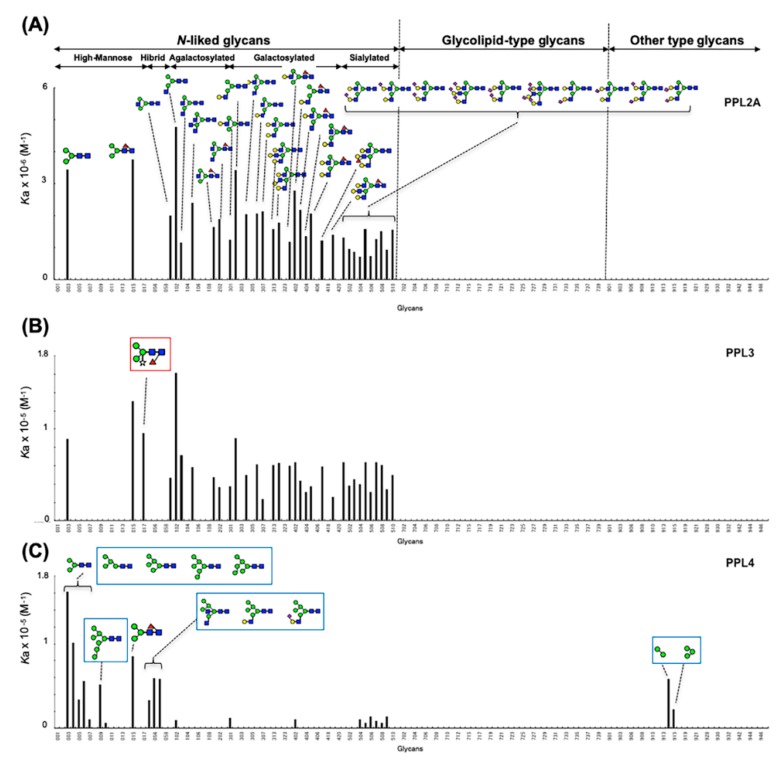
Bar graph representation of affinity constants (*K*_a_) of PPL2A (**A**), PPL3s (**B**), and PPL4 (**C**) toward N-linked glycans, glycolipid-type glycans, and other glycans. Small Arabic figures at the horizontal axes correspond to sugar numbers indicated in Supplementary Materials Figure S6, which are classified into high-mannose-type, hybrid-type, agalacto-type, galactosylated-type and sialylated-type N-linked glycans, glycolipid-type glycans, and others. Glycans in red and blue boxes are those showing the different specificities for PPL3s and PPL4, respectively, compared with PPL2A.

Figure 4 shows the bar graph of *K*_a_ values of PPL2A, PPL3s, and PPL4 against 130 PA glycans (Supplementary Materials Figure S6). Compared with PPL2A and PPL3s, both PPL2A and PPL3s possess quite similar binding profiles, except for Man_3_Fuc_1_Xyl_1_GlcNAc_2_ oligosaccharide (017) (Figure 4A,B), which is unique to plant glycoproteins [29], although they showed no affinity toward glycolipid-type glycans and other glycans. PPL2A and PPL3s showed affinities to agalactosylated-type glycans (101–103, 105, 201, and 202 in Supplementary Materials Figure S6), galactosylated-type glycans (301, 302, 304, 306, 307, 313, 314, 401–405, 410, and 419), and sialylated-type glycans (501–510), although they can also bind to core penta saccharide of N-glycans, 003 and 015, but not for other high-mannose-type and hybrid glycans. PPL2A showed much stronger binding affinity (~100 times) than PPL3s. On the other hand, PPL4 showed a different carbohydrate-binding profile compared with PPL2A and PPL3s; that is, PPL4 showed affinity to high-mannose-type glycans (003–006, 009, 010, 015) and hybrid-type glycans (053, 056, 057), but not agalactosylated-and galactosylated-type glycans (except for 102, 301, and 402) (Figure 4). PPL4 showed no affinity toward glycolipid-type glycans or others, except for 914 and 915 containing Manα1–6Man structure but not Manα1–3Man (913) (Figure 4C). These results suggest that PPL4 recognize Manα1–6Man moiety in carbohydrate structures.

Thus, the carbohydrate-binding properties of PPL2A and PPL3s can be summarized as follows: (a) PPL2A and PPL3s possess similar binding profiles, except for Man_3_Fuc_1_Xyl_1_GlcNAc_2_ oligosaccharide; (b) PPL2A and PPL3s show affinities for agalactosylated-type and galactosylated-type N-linked glycans, except for bisecting GlcNAcβ1–4 Man containing glycans and four GlcNAc branched N-glycans; and (c) they also show affinity for sialylated-type N-glycan, but not for glycolipid-type glycans. On the other hand, PPL4 shows the following properties: (a) PPL4 binds to high-mannose- and hybrid-type N-linked glycans, but not agalactosylated-and galactosylated-type glycans; (b) PPL4 shows high affinity against Manα1-6Man structure but not Manα1–3Man. All PPL3s and PPL4 showed no affinity against glycolipid-type glycans.

## 3. Discussion

Two novel lectins, PPL3s (3A, 3B, 3C) and PPL4, were isolated from the secreted fluid of mantle of *Pteria penguin* pearl shells and characterized. The amino acid sequences of PPL3 and PPL4 subunits showed sequence homologies with PPL2A and PPL2B, which were previously isolated from the mantle of *Pteria penguin* [23]. PPL3 and PPL4 subunits also share homologous sequences (20–30%) with ZG-16p and jacalin (Figure 3), indicating that PPL3s and PPL4, as well as PPL2s, belong to the jacalin-related lectin (JRL) family. Multiple sequence alignment of PPL3 and PPL4 subunits with JRLs revealed that the structural elements for the carbohydrate recognition domain (CRD) were conserved among PPLs and JRLs, including the GG loop (Gly28/Gly31) and the binding loop (Gly149/Asp153) at the top of β-prism fold [30,31,32], although PPLs have low sequence identity with other JRLs (Figure 2). These results suggest that PPL3s and PPL4 possess one CRD per subunit, and are active dimers, in addition to PPL2s and other JRLs. More recently, we have determined 3D structure of PPL3s [24]. It revealed the recognition residues at the CRD interacted with the Man pyranose ring and created a hydrogen bond network with OH of Man in PPL3s, including Gly31, Asn77, Trp81, Tyr108, Arg151, and Asp153. Among them, Gly31 at the GG loop and Asp153 at the carbohydrate-binding domain were conserved in all PPLs, while the amino acid residues Asn77 and Trp81 on helix 2, and Tyr108 at the recognition loop that conserved between PPL2A and PPL3s, were replaced by Ser77, Asp81, and Phe108 in PPL4, respectively (Figure 2 and Figure 3). Especially, PPL4α and β subunits contained the inserted extra three residues on helix 2. These different residues on helix 2 and the recognition loop might cause the different carbohydrate-binding specificities between PPL2A/PPL3s and PPL4. The hemagglutinating inhibition assay showed that PPL3s have sugar-binding specificities against trehalose and isomaltose similar to that of PPL2A, while PPL4 showed a strong affinity for *N*-acetyl-D-glucosamine and its polymer, chitin oligomer, and weak affinity for trehalose (Table 1). Furthermore, FAC analysis data also showed similar carbohydrate-binding properties of PPL2A and PPL3s, except for Man_3_Fuc_1_Xyl_1_GlcNAc_2_ oligosaccharide (Figure 4), although their primary sequences—including inter-subunit disulfide bond—were quite different. PPL2A and PPL3s mainly recognize agalactosylated- and galactosylated-type glycans, while PPL4 binds to high-mannose-and hybrid-type N-linked glycans.

PPL2A, 2B, and PPL4 contain one interchain disulfide bond at the N-terminus (head-to-head); only PPL3s contain half-cystine for an interchain disulfide bond at the C-terminus (tail-to-tail). The 3D structure of PPL3s also confirmed a tail-to-tail dimer structure by forming a unique inter-subunit disulfide bond at the C-termini, and that the N-terminal residues of PPL3s were found in both pyroglutamate (pGlu-Val) and glutamate (Glu-Ile) forms, which can be explained by the post-translational modification of PPL3 isoforms implied from the discrepancy between amino acid and gene sequences. From the 3D structure of PPL3s, it was found that the C-terminal and N-terminal regions are spatially in close proximity, within 5.8 Å, and located on the opposite side of the CRD, indicating that the different position of the inter disulfide bond at the N-terminal or the C-terminal may have little or no effect on the carbohydrate-binding specificities of PPLs.

**Figure 5:**
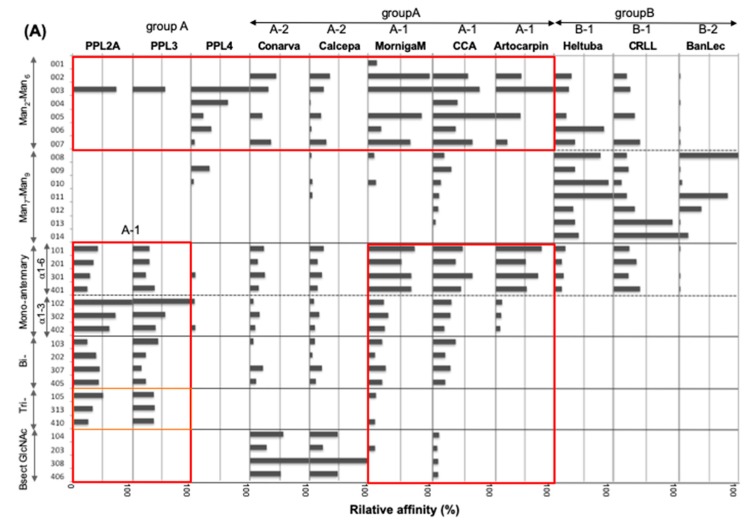

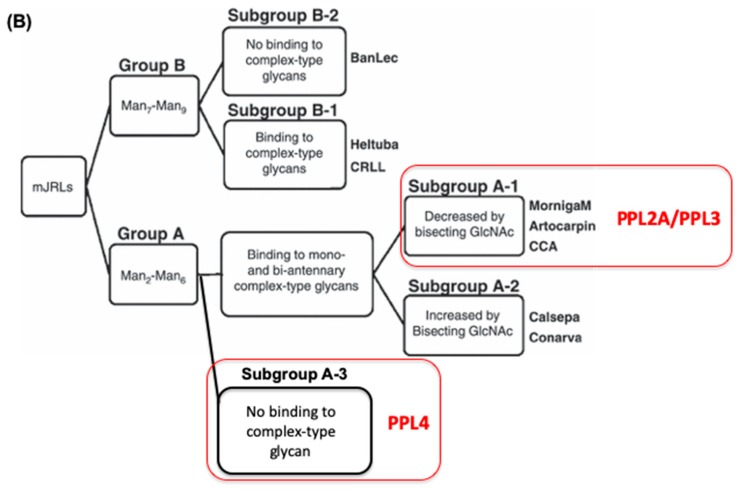
Bar graph representation of the relative affinity (%) of PPL2A, PPL3s, and PPL4 with another eight mJRLs towards selected N-linked glycans (**A**) and classification of mJRLs by sugar-binding specificities (**B**). Data for Conarva, Calcepa, MornigaM, CCA, Artocarpin, Heltuba, CRLL, and BanLec are cited from Nakamura-Tsuruta et al. (2008) [33]. The numbers on the left correspond to the numbers of selected glycans listed in Supplementary Materials Figure S6. Groups A (A-1, A-2) and B (B-1, B-2) show the classification of mJRLs based on FAC analysis.

Previously, Nakamura-Tsuruta et al., (2008) reported the functional classification of jacalin-related lectins based on their detailed oligosaccharide specificities, made by the FAC system, that clearly divided mJRLs into two major groups on the basis of their specificity towards high-mannose-type glycans (groups A and B), each of which was further divided into two subgroups based on the preference for complex-type glycans (Figure 5A) [33]. According to the sugar-binding specificities, PPL2A and PPL3s can be classified into group A (subgroup A-1), the same as MornigaM, Artocarpin, and *Cancer antennarius* lectin (CCA), which bind to complex-type glycans and are reduced by a bisecting GlcNAc. On the other hand, PPL4 is also classified into group A, but cannot be classified into any subgroups because PPL4 shows no binding to complex-type glycans like a BanLec (subgroup B-2) (Figure 5), indicating that PPL4 is a new type of JRL. Thus, JRLs in *P. penguin* (PPL2A, PPL3s, and PPL4) show very unique carbohydrate-binding abilities different from one another and other JRLs. Secretory fluid from the mantle of pearl shells contains many shell components, which play significant roles in shell calcification, such as matrix proteins and carbohydrates like chitin [34]. In particular, beta-chitin is well known as a key component of the extracellular matrix of mollusk shells produced by chitin synthases [35,36,37]. In mollusk shells, beta-chitin is highly ordered at the molecular level and is responsible for the interlamellar structure, due to its silk-like protein and acidic proteins [38]. Recently, it has been reported that polysaccharides containing high levels of carboxylate and sulfate also regulate (promote) growth and morphology of calcite crystals [39]. In the present study, we identified PPL4, of which carbohydrate specificities include chitin specific affinity. Previously, we found that trehalose abundant in *P. penguin* mantle had an increasing effect of crystal formation with PPL2A [23]. Considering the crucial roles of polysaccharides in crystallization, diverse carbohydrate-binding proteins such as PPLs are thought to be potential key players in biomineralization. Further investigations are needed to elucidate the biomineralization mechanisms of PPLs as matrix proteins.

In conclusion, we present new lectins termed PPL3s and PPL4, in addition to PPL2A, which contribute to the biomineralization processes of *P. penguin* nacreous shells with different carbohydrate recognition profiles. PPL2A and PPL3s recognize agalactosylated-and galactosylated-type glycans, while PPL4 binds to high-mannose-and hybrid-type N-linked glycans and chitin oligomer. These observations highlight the unique functions of this lectin family and their specific carbohydrates, which work collaboratively in mollusk shell formation.

## 4. Materials and Methods

### 4.1. Materials

Large-winged pearl shells (*Pteria penguin*, 6 years old) were provided by Amami South Sea & Mabe Pearl Co. Ltd. (former Amami-branch, Tasaki & Co. Ltd.), Kagoshima, Japan. Mantle and its secretory fluid were collected and stored at −80 °C or −30 °C until use, respectively. A POROS^®^ HS column was purchased from Applied Biosystems Thermo Fisher Scientific (Waltham, MA, USA). Resource S and HiTrap NHS-activated columns were purchased from GE Healthcare UK Ltd. (Amersham Place, Buckinghamshire, UK). TSKgel ODS-120T, COSMOSIL Protein-R, and CAPCELL PAK columns were purchased from Tosoh (Tokyo, Japan), SHISEIDO (Tokyo, Japan) and Nacalai tesque (Kyoto, Japan), respectively. *Achromobacter* protease I and *Staphylococcus aureus* V8 protease were purchased from Wako Pure Chemical Ind. (Osaka, Japan). Trehalose was purchased from Hayashibara (Okayama, Japan). PA-oligosaccharides were purchased from TaKaRa Bio Co. (Kyoto, Japan). All other reagents were of the purest grade commercially available.

### 4.2. Isolation of Lectins from the Secretory Fluid of Mantle

Secretory fluid of mantle was dialyzed against desalinized water and lyophilized. The lyophilizate was dissolved in distilled water and centrifuged (3000× *g*). The supernatant was fractionated by cation-exchange chromatography on a POROS HS column equilibrated with 10 mM MES/HEPES buffer (pH 6.5), and eluted with a linear gradient of 0 to 1 M NaCl (Figure 3). The eluted fractions, named HSA, HSB, HSC, HSD, and HSE, sequentially, were dialyzed against water and lyophilized. HSA and HSE fractions showing agglutinating activities against rabbit erythrocytes were further purified by cation-exchange chromatography on a Resource S column pre-equilibrated with 20 mM MES buffer (pH 7.0), and eluted with a linear gradient of 0 to 2 M NaCl in the same buffer, respectively.

Molecular masses of PPL3A, 3B, 3C, PPL4, and their subunits were determined by sodium dodecyl sulfate-polyacrylamide gel electrophoresis (SDS-PAGE) and matrix-assisted laser desorption ionization time of flight (MALDI-TOF) mass spectrometry, respectively. SDS-PAGE was performed using a 15% separating gel in the presence or absence of 2-mercaptoethanol, and protein bands were stained by Coomassie Brilliant Blue R-250 (Nacalai tesque). For MALDI-TOF mass spectrometry, aqueous solutions of protein samples were diluted 2-fold with 0.1% trifluoroacetic acid (TFA) containing sinapinic acid (1 mg/mL) as matrix. One microliter of mixture was deposited on a plate and air-dried. MALDI-TOF/TOF 5800 mass spectrometer (Sciex, Framingham, MA, USA) was equipped in linear mode with an acceleration voltage of 25 kV. Mass spectra were obtained as averages of 100 laser shots, and several independent MALDI-TOF mass measurements were made for each sample to evaluate reproducibility. Insulin (Mr 5733.5) (bovine pancreas, Sigma-Aldrich, St. Louis, MO, USA) and myoglobin (Mr 16,949.5) (horse heart, Sigma-Aldrich) were used for external calibration.

### 4.3. Amino Acid Sequences of PPL3 and PPL4 Subunits

The amino acid sequence of each subunit for PPL3A, 3B, 3C, and PPL4 was determined via Edman sequencing of peptides generated by digestion of *S*-carboxamidomethyl (CAM) proteins with various proteases and cDNA sequencing. PPL3A, 3B, 3C (1.0 or 2.0 mg), and PPL4 (800 μg) were *S*-carboxamidomethylated as previously mentioned. The appropriate amount of protein was reduced with 4 mM dithiothreitol at 50 °C for 15 min in the presence of urea. According to the method of Stone et al. [40], the reaction mixture was treated with 8 mM iodoacetamide at room temperature for 30 min in the dark to convert freshly-generated cysteine residues into CAM-cysteine.

CAM-PPL3s were digested with endoproteinase Lys-C (Wako, Osaka, Japan) (substrate/enzyme (S/E), 200:1) and *Staphylococcus aureus* V8 protease (Wako) (S/E, 100:1 or 50:1) according to the manufacturer’s recommendations, respectively. CAM-PPL4 was digested with *Achromobacter* protease I after separation of subunits by reversed-phase chromatography. Each digest was separated by reversed-phase HPLC on a TSKgel ODS 120T column (ø 4.6 × 250 mm) (Tosoh, Tokyo, Japan) or COSMOSIL^®^ Protein-R column (ø 4.6 × 250 mm) (Nacalai tesque, Kyoto, Japan) using a linear gradient of acetonitrile in 0.1% TFA. Amino acid sequences of peptides were determined by protein sequencer (PPSQ-10, Shimadzu, Japan) and MALDI-TOF mass spectrometer.

Total RNA was extracted from *P. penguin* mantle by the guanidium thiocyanate/phenol/chloroform method, and poly (A)+ RNA was purified using a Micro-Fast Track mRNA Isolation kit (Invitrogen, Carlsbad, Calif.). A cDNA library was constructed from poly (A)+ RNA using a Marathon cDNA Amplification kit (Clontech, Palo Alto, Calif., USA), as previously reported [22,23]. To amplify cDNAs of PPL3 and PPL4 subunits, we designed the sense and anti-sense gene-specific primers (GSPs) based on the partial amino acid sequences of PPL3s and PPL4, respectively (Supplementary Materials Table S1). PCR was performed with KOD polymerase (Toyobo Co., Tokyo, Japan) for 30 cycles of repeating denaturation at 96 °C for 30 s, annealing at 45 °C for 30 s, and extension at 68 °C for 1 min using cDNA library as a template and primers. Full-length cDNA sequences of PPL3 (and) and PPL4 (and) subunits were determined by 3′-and 5′-rapid amplification of cDNA ends (RACE) with GSP for each subunit (Supplementary Materials Table S1) and adaptor primer 1 (AP1), 5′-CCATCCTAATACGACTCACTATAGGGC-3′. Each amplified cDNA fragment was subcloned into a pCR–Blunt II-TOPO plasmid (Invitrogen), and the nucleotide sequence (Appendix A) was determined by Applied Biosystems Model 310 DNA sequencers. The nucleotide sequences of cDNAs encoding PPL3α, PPL3β, PPL4α, and PPL4β subunits were deposited in DDBJ/Genbank database under accession numbers AB425240.1, LC334154, AB425241.1, and AB425242.1, respectively.

The amino acid sequences of PPL3s and PPL4 (α and β subunits) were compared with other jacalin-related lectins, Jacalin: Galactose-binding lectin from *A. integer* (AAA32677) and human-ZG16 (BAC20361) using a multiple alignment computed by the Clustal W program [24].

### 4.4. Sugar-Binding Specificity

Hemagglutination inhibition assays for PPL3s and PPL4 were conducted using rabbit erythrocytes. Samples were serially diluted with 50 μL of 0.15 M NaCl on microtiter plates and mixed with equal volume of 2% rabbit erythrocyte suspension for 1 h. Hemagglutination activity was defined as the titer value of maximum dilution with positive agglutination of 2% rabbit erythrocytes. The inhibitory effects of saccharides on hemagglutination were also assayed. Saccharide solutions (25 μL) were diluted 2-fold in series on microtiter plates and incubated with 25 μL of lectin solution with hemagglutination titer values of 2^−3^ for 15 min. The rabbit erythrocytes suspension (2%, 50 μL) was added to mixture and incubated for another 30 min. The inhibitory activities were estimated by the minimum concentration of sugar needed to cause negative hemagglutination.

Furthermore, frontal affinity chromatography (FAC) was performed by using the automated FAC system [26]. Briefly, purified PPL2A, PPL3s, and PPL4 were dissolved in 10 mM phosphate buffer pH 7.4, containing 0.7% NaCl and bound to NHS-activated Sepharose according to the manufacturer’s instructions, respectively. After deactivating the excess NHS by 1 M monoethanolamine and washing, lectin-conjugated Sepharose was suspended in 10 mM Tris-HCl (pH 7.6) containing 150 mM NaCl. Slurry was packed into a miniature column (ø 2 × 10 mm, bed volume, 31.4 μL). The amount of bounded protein was determined by measuring of uncoupled protein by Bradford method. For determination of effective ligand content (B_t_) in the lectin column, concentration-dependent analysis using 1M2M-5NC-Asn (Fmoc) for PPL2A and PPL3s, and Manα1-pNP for PPL4, was conducted. Woolf–Hofstee-type plots, i.e., (V–V_0_) vs. (V–V_0_) [A]_0_, were made to determine B_t_ and *K*_d_ values from the intercept and the slope of the fitted curve, respectively. After equilibrium of miniature columns had been obtained, PA-oligosaccharides (2.5 nM) dissolved in TBS were successively injected into a pair of lectin columns by the auto-sampling system. Elution of PA-oligosaccharides (listed in Supplementary Materials Figure S6) was monitored by fluorescence (excitation and emission wavelengths of 310 and 380 nm, respectively). For FAC analysis, retardation of the elution front of each oligosaccharide relative to that of an appropriate standard oligosaccharide, i.e., V–V_0_, was determined. *K*_d_ values for dissociation of lectin and oligosaccharide were obtained from V–V_0_ and B_t_, according to the basic equation of FAC, *K*_d_ = B_t_/(V-V_0_)-[A]_0_, where B_t_ is the effective ligand content (expressed in mol), and [A]_0_ is the initial concentration of PA-oligosaccharide. This equation can be simplified to Eqn: *K*_d_ = B_t_/(V − V_0_), where [A]_0_ is negligibly small (e.g., <10^−8^ M) compared with *K*_d_ (e.g., >10^−6^ M).

## Figures and Tables

**Table 1:** Carbohydrate-binding specificities of PPL3s and PPL4 compared with PPL2A and 2B.

Saccharides/Glycoproteins		PPL2A ^a^	PPL2B ^a^	PPL3	PPL4
		mM ^b^			
D-Glucose		>250	>250	200	25
D-Fructose		250	>250	50	50
D-Mannose		>250	>250	100	25
D-Glucosamine		>250	>250	>200	200
*N*-Acetyl-D-glucosamine		250	>250	>200	6.25
Trehalose	Glc(α1-1α)Glc	7.8	>250	25	100
Kojibiose	Glc(α1-2α)Glc	>250	125	>200	100
Maltose	Glc(α1-4α)Glc	>250	>250	>200	200
Isomaltose	Glc(α1-6α)Glc	7.8	>250	12.5	>200
Others ^c^		>250	>250	>200	>200
		%(*w*/*v*) ^b^			
Chitin oligomers	GlcNAc(β1-4β)GlcNAc	>0.25	>0.25	>0.25	0.125
Chitosan oligomers	GlcNH_2_(β1-4β)GlcNH_2_	>0.25	>0.25	>0.25	>0.25
Fetuin		>0.25	0.25	0.025	0.025
Asialo fetuin		0.078	0.078	0.0125	0.025
Other ^d^		>0.25	>0.25	>0.25	>0.25

^a^ Data from previous work (Naganuma et al., 2014 [23]). ^b^ Minimum concentration of saccharides (mM), polysaccharides and glycoproteins (%(*w*/*v*)) required for complete inhibition. ^c^ d-Galactose, d-Galactosamine, *N*-Acetyl-d-galactosamine, d-Xylose, l-Fucose, l-Arabinose, l-Rhamnose, Cellobiose. ^d^ Asialomucin type I, asialomucin type II, heparin.

## Supplementary Materials

Supplementary Materials can be found at https://www.mdpi.com/1422-0067/20/18/4629/s1. Figure S1. Peptide maps of CAM-PPL3A, 3B, and 3C digested by *Achromobacter* protease I (**A**) and *Staphylococcus aureus* V8 protease (**B**); Figure S2. Nucleotide sequences of cDNAs and corresponding amino acid sequences of PPL3 subunits; Figure S3. Separation of CAM-PPL4 α and β subunits by reversed-phase HPLC; Figure S4. Peptide maps of CAM-PPL4 α (**A**) and β (**B**) subunits digested by *Achromobacter* protease I; Figure S5. Nucleotide sequences of cDNAs and corresponding amino acid sequences of PPL4 (**A**) and (**B**) subunits; Figure S6. Schematic representation of oligosaccharide structures; Table S1. Amino acid sequences and masses of the peptides generated by cleavage of CAM-PPL3B with *Achromobacter* protease I (**A**) and *S. aureus* V8 protease (**B**); Table S2. Amino acid sequences and masses of the peptides generated by cleavage of CAM-PPL4 (**A**) and (**B**) subunits with *Achromobacter* protease I; Table S3. Properties of lectin-immobilized columns used for FAC analysis.

## Abbreviations

CAM	*S*-carboxamidomethylated
CCA	Cancer antennarius lectin
CRD	carbohydrate recognition domain
FAC	frontal affinity chromatography
GlcNAc	*N*-acetyl-D-glucosamine
HPLC	high-performance liquid chromate
JRL	jacalin-related lectin
PPL	*Pteria penguin* lectin
SDS-PAGE	sodium dodecyl sulfate polyacrylamide gel electrophoresis
SEM	scanning electron microscopy

## Appendix A

Accession Numbers: The nucleotide sequence data reported in this study were deposited in the DDBJ, EMBL, and GenBank databases under accession numbers AB425240.1, LC334154, AB425241.1, and AB425242.1 for PPL3α, PPL3β, PPL4α, and PPL4β, respectively.
