# Supplementary material for: Glycan Binding Profiling of Jacalin-Related Lectins from the Pteria Penguin Pearl Shell
Source: Int J Mol Sci. 2019 Sep 18;20(18):4629. doi: 10.3390/ijms20184629 (PMC6769917; doi:10.3390/ijms20184629)
Supplement: Supplementary file 1 [file ijms-20-04629-s001.pdf]

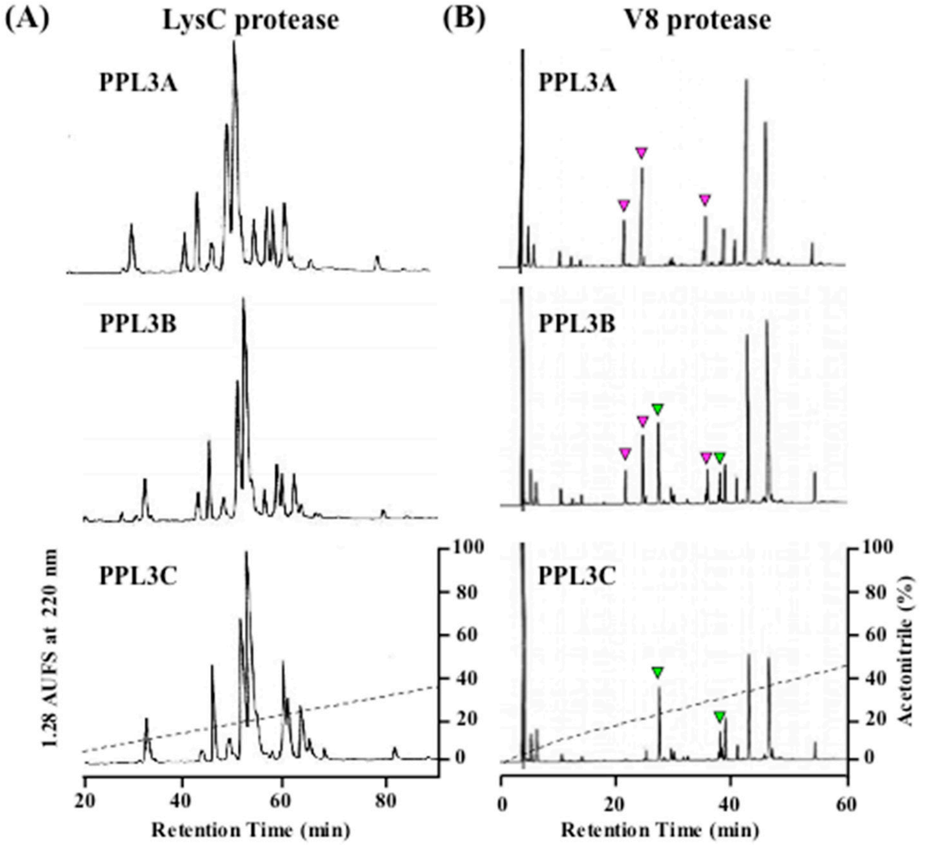

Supplementary Figure S1. Peptide maps of CAM-PPL3A(α+α), 3B(α+β), and 3C(β+β) digested by *Achromobacter* protease I (**A**) and *Staphylococcus aureus* V8 protease (**B**). Peptides were separated by reversed-phase HPLC on COSMOSIL® Protein-R colomn (ø 4.6 × 250 mm) using a linear gradient of acetonitrile in 0.1% trifluoroacetic acid. Common peaks between *α* and β subunits were marked by magenta and green-colored arrow heads, respectively.

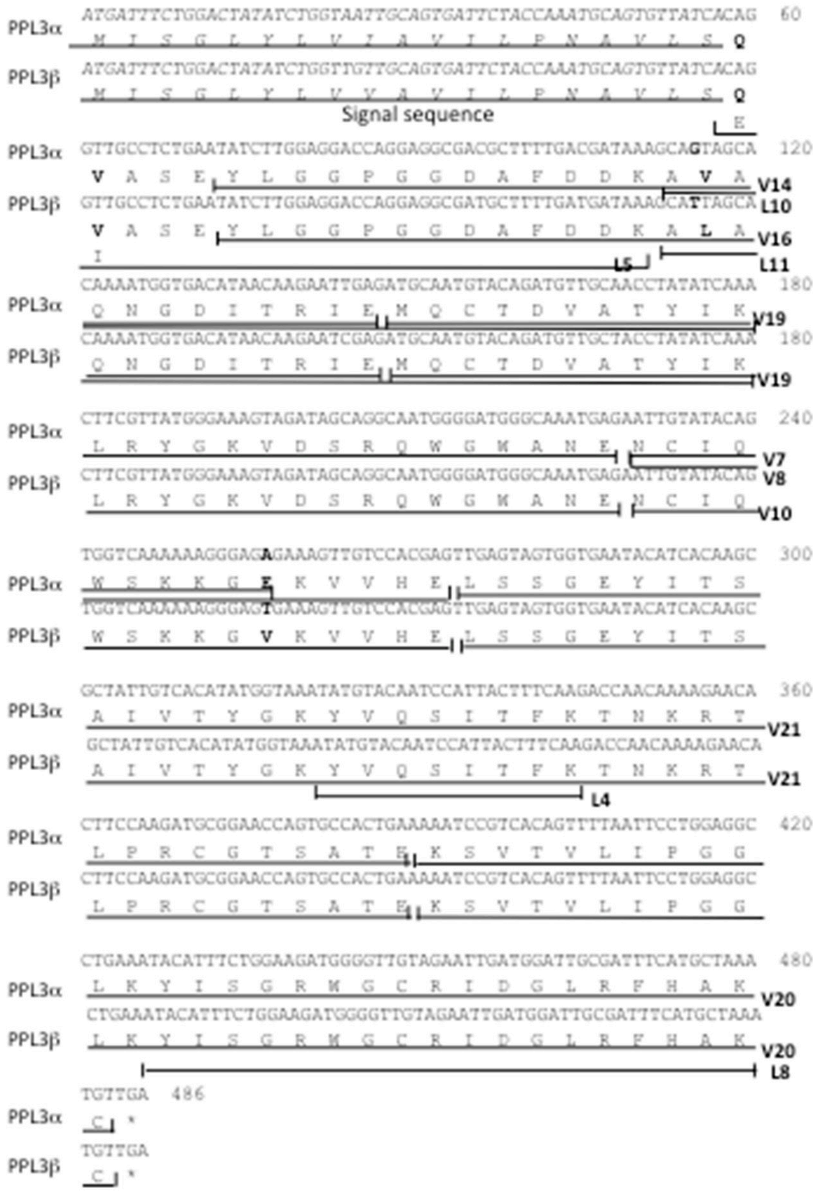

Supplementary Figure S2. Nucleotide sequences of cDNAs and the corresponding amino acid sequences of PPL3 subunits. Nucleotide residues and amino acid residues different from PPL3α and β subunits are indicated by bold characters, respectively. Peptide fragments generated by digestion with *Achromobacter* protease I (L) and *S. aureus* V8 protease (V), respectively, are indicated by lines. Italic letter with underline indicates the signal peptide region. Asterisk indicates the stop codon.

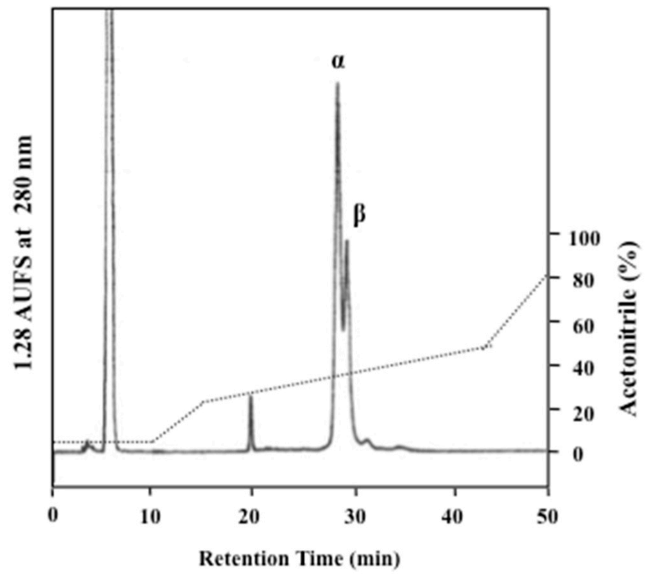

Supplementary Figure S3. Separation of CAM-PPL4 α and β subunits by reversed-phase HPLC. Separation of CAM-PPL4 subunits was conducted by HPLC on a CAPCELL PAK (C8) colomn (ø 4.6 × 150 mm) using graded linear gradient of acetonitrile in 0.1 % TFA.

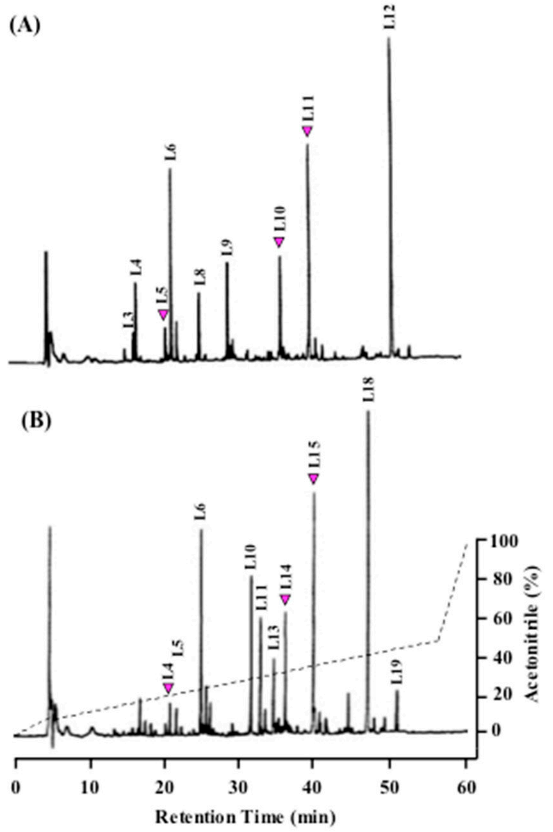

Supplementary Figure S4. Peptide maps of CAM-PPL4 α (A) and β (B) subunits digested by *Achromobacter* protease I. Peptides were separated by reversed-phase HPLC on COSMOSIL® Protein-R colomn (ø 4.6 × 250 mm) using a linear gradient of acetonitrile in 0.1% trifluoroacetic acid. Common peaks between α and β subunits were marked by magenta arrow heads.

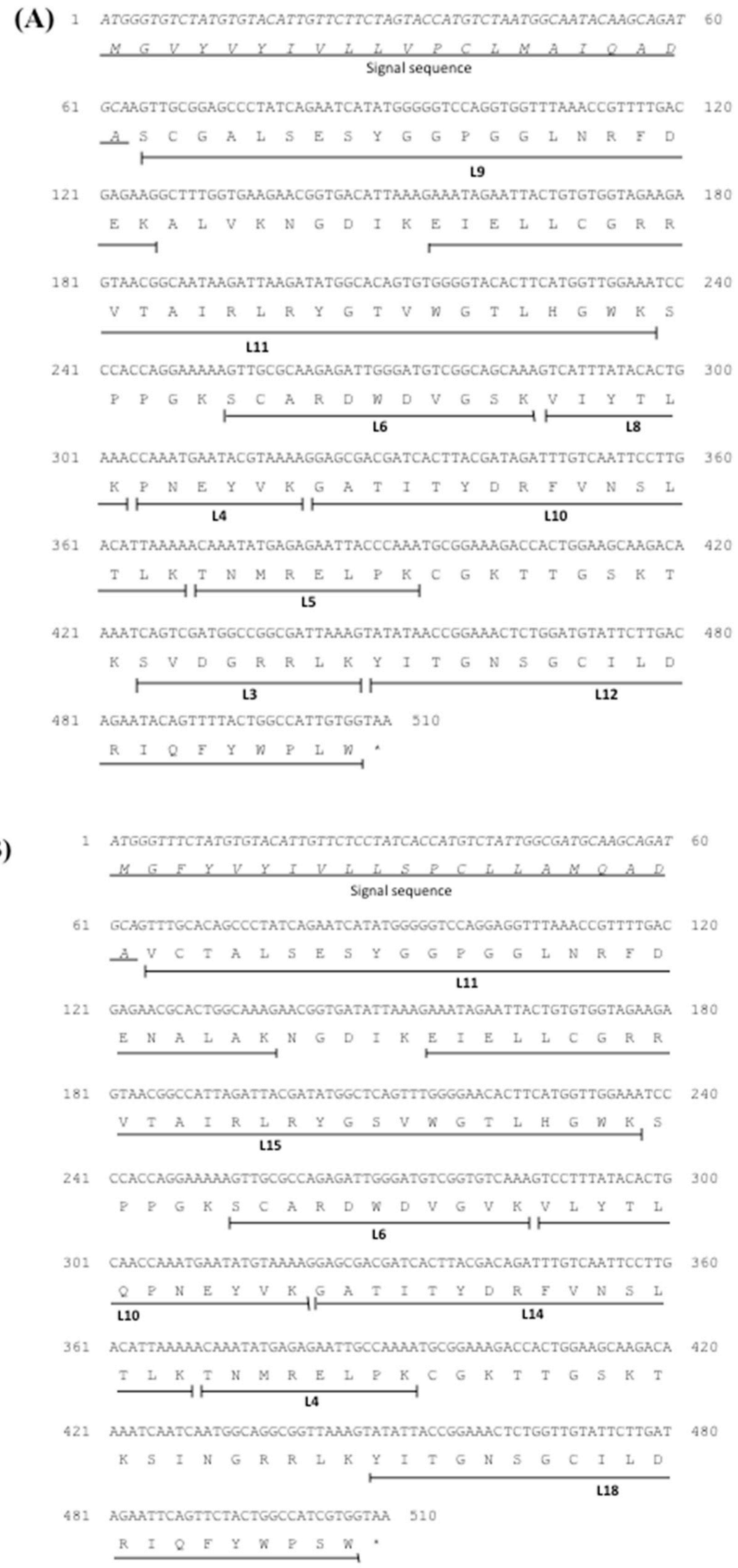

Supplementary Figure S5. Nucleotide sequences of cDNAs and the corresponding amino acid sequences of PPL4 α (A) and β (B) subunits. Peptide fragments generated by *Achromobacter* protease I (L) digestion are indicated by lines. Italic letter with underline indicates the signal peptide region. Asterisk indicates the stop codon.

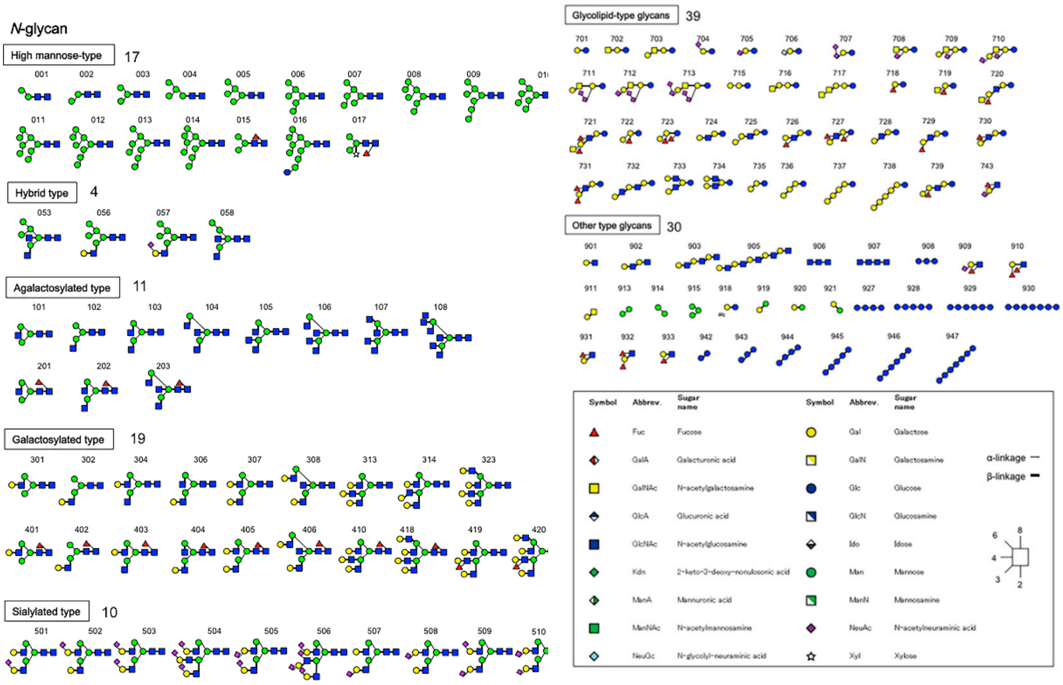

Supplementary Figure S6. Schematic representation of oligosaccharide structures. Note that the reducing terminal is pyridylaminated for FAC analysis. Symbols used to represent pyranose rings of monosaccharides are shown in the box at the bottom. Anomeric carbon, i.e. position 1, is placed at the right side, and 2, 3, 4 are placed clockwise. Thin and thick bars represent α-linkage and β-linkage, respectively.

Supplementary Table S1. The amino acid sequences and masses of the peptides generated by cleavage of the CAM-PPL3B with *Achromobacter* protease I (**A**) and *S. aureus* V8 protease (**B**).

(A) *Achromobacter* protease I

Fragment number	Amino acid sequences		Molecular mass (m/z)	
			Calculated	Observed
L4	YVQSITFK		984.55	987.10
L5	EIASEYLGGPGGDAFDDK	[N terminus]	1839.84	1844.19
L8	YISGRWGCRIDGLRFHAK		2192.18	2196.43
L9	VDSRQWGWANENCIQWSK		2264.09	2269.28
L10	A**V**AQNGDITRIEMQCTDVATYIK		2597.33	2601.46
L11	A**L**AQNGDITRIEMQCTDVATYIK		2611.34	2616.13

(B) *S.aureus* V8 protease

Fragment number	Amino acid sequences	Molecular mass (m/z)	
		Calculated	Observed
V7	NCIQWSKKG**E**	1250.64	1250.01
V8	NCIQWSKKG**E**KVVHE	1842.97	1842.29
V9	ITFKTNKRTLPRCGTSATE	2182.18	2181.85
V10	NCIQWSKKG**V**KVVHE	1813.00	1812.06
V14	YLGGPGGDAFDDKA**V**AQNGDITRIE	2579.24	2579.64
V16	YLGGPGGDAFDDKA**L**AQNGDITRIE	2593.25	2593.96
V17	VATYIKLRYGKVDSRQWGWANE	2640.37	2640.23
V19	MQCTDVATYIKLRYGKVDSRQWGWANE	3276.61	3276.47
V20	KSVTVLIPGGLKYISGRWGCRIDGLRFHAKC	3546.99	3545.02
V21	LSSGEYITSAIVTYGKYVQSITFKTNKRTLPRCGTSATE	4329.26	4325.63

Common peptides between PPL3A (α+α) and PPL3B (α+β), and between PPL3B (α+β) and PPL3C (β+β) are indicated by magenta and green boxes, respectively.

Supplementary Table S2. The amino acid sequences and masses of the peptides generated by cleavage of the CAM-PPL4α (A) and β (B) subunits with *Achromobacter* protease I.

(A) PPL4α

Fragment number	Amino acid sequences		Molecular mass (m/z) Calculated	Observed
L3	SVDGRRLK		929.56	932.82
L4	PNEYVK		748.40	773.46
L5	TNMRELPK		987.54	991.19
L6	SCARDWDVGSK		1280.62	1283.11
L8	VIYTLK		735.48	760.08
L9	SCGALSESYGGPGGLNRFDEK	[N terminus]	2201.05	2203.56
L10	GATITYDRFVNSLTLK		1797.99	1802.24
L11	EIELLCGRRVTAIRLRYGTVWGTLHGWK		3340.88	3344.89
L12	YITGNSGCILDRIQFYWPLW		2502.28	2506.58

(B) PPL4β

Fragment number	Amino acid sequences		Molecular mass (m/z) Calculated	Observed
L4	TNMRELPK		987.54	990.42
L6	SCARDWDVGVK		1292.66	1295.40
L10	VLYTLQPNEYVK		1465.81	1469.29
L11	VCTALSESYGGPGGLNRFDENALAK	[N terminus]	2626.31	2630.86
L14	GATITYDRFVNSLTLK		1797.99	1802.24
L15	EIELLCGRRVTAIRLRYGSVWGTLHGWK		3326.86	3331.88
L18	YITGNSGCILDRIQFYWPSW		2476.23	2478.06

Common peptides between PPL4α and β subunits are indicated by magenta boxes.

Supplementary Table S3. Properties of lectin-immobilized columns used for FAC analysis.

Lectin name	Amount of Immobilized lectin (mg/ml gel)	Bt (nmol)	*Kd* (M)	R^{2} [a]	Used carbohydrate
PPL2A	0.05	0.02	2.0×10^{-7}	0.996	1M2M-5NC-Asn Fmoc
PPL3	0.5	0.63	3.01×10^{-5}	0.985	1M2M-5NC-Asn Fmoc
PPL4	1.0	0.98	2.0×10^{-5}	0.996	ManαpNP

[a] the coefficient of determination quantified the degree of linear correlation obtained from a Woolf-Hofstee-type plot in each concentration-dependent analysis. B_t and K_d values were calculated from those determined by concentration-dependent analysis.
